# Dandelion (*Taraxacum* Genus): A Review of Chemical Constituents and Pharmacological Effects

**DOI:** 10.3390/molecules28135022

**Published:** 2023-06-27

**Authors:** Min Fan, Xiao Zhang, Huaping Song, Yakong Zhang

**Affiliations:** 1 Department of Traditional Chinese Medicine, Gansu Medical College, Pingliang 744000, China; ndqsfs@163.com; 2 Sanitation Test Center, Pingliang Center for Disease Control and Prevention, Pingliang 744000, China; zhangxiao5922017@126.com

**Keywords:** *Taraxacum* genus, dandelion, phytology, plant active ingredients, pharmacology, toxicity

## Abstract

Dandelion (*Taraxacum* genus) is a perennial herb belonging to the Asteraceae family. As a well-known and extensively studied genus, dandelion comprises numerous species. Some species have been widely used in both complementary and alternative medicine to clear heat, detoxify, activate blood circulation, dispel stasis, and discharge urine. Multiple pharmacological studies have highlighted its therapeutic potential, including anti-bacterial, anti-oxidant, anti-cancer, and anti-rheumatic activities. Furthermore, bioactive compounds associated with these effects include sesquiterpenoids, phenolic compounds, essential oils, saccharides, flavonoids, sphingolipids, triterpenoids, sterols, coumarins, etc. Based on recent studies about the *Taraxacum* genus, the present review critically evaluates the current state of dandelion utilization and summarizes the significant roles of dandelion and its constituents in different diseases. We also focus on the reported phytology, chemical composition, pharmacology, and toxicity of dandelion, along with the main possible action mechanisms behind their therapeutic activities. Meanwhile, the challenges and future directions of the *Taraxacum* genus are also prospected in this review, thus highlighting its pharmaceutical research and practical clinical applications.

## 1. Introduction

Dandelion (*Taraxacum* genus), named “Pugongying” in China, is a perennial plant belonging to the Asteraceae family. It has a complex classification, comprising over three hundred species [1]. In Asia, the *Taraxacum* genus is widely cultivated and also found wild in most parts of China, North Korea, Mongolia, and Russia [2]. It grows in temperate regions globally, including on lawns, on roadsides, on disturbed banks and shores of waterways, and in other areas with moist soils.

**Figure 1:**
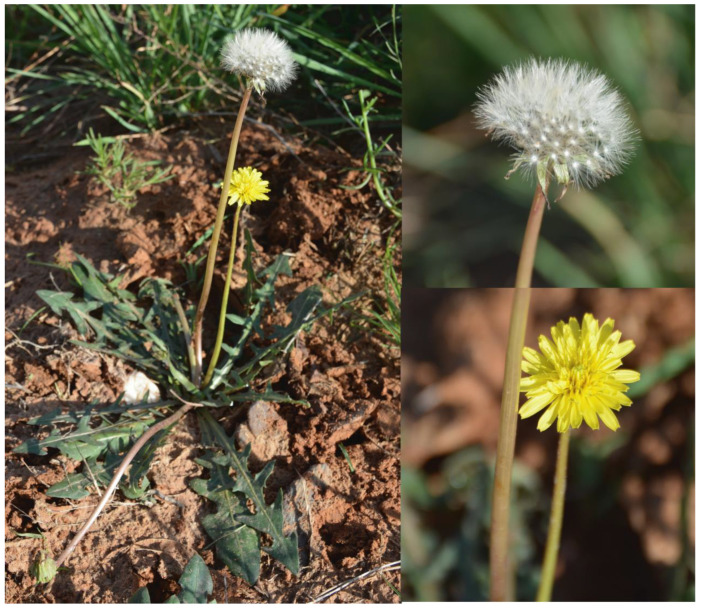
The morphology of *Taraxacum officinale*.

As an edible medicinal herb and vegetable, dandelion (*Taraxacum* genus) has long been utilized in traditional medicine, folk remedies, and substitution therapies in many countries to treat diverse diseases (Figure 1) [3]. *Taraxacum* genus as a drug was first used to treat liver and spleen diseases in Arabian medicine. In the 16th century, the German botanist Fuchs discovered that *Taraxacum* can be used to treat gout, diarrhea, blisters, and spleen and liver diseases. It has been used as a common drug for detoxification, swelling, and lactation since the 16th century in China. Since the 19th century, several authors have relied on the existing traditional knowledge to provide scientific explanations about how *Taraxacum* works on diseases and their symptoms [4]. *Taraxacum* can be used as diuretics, antioxidants, bile agents, anti-inflammatory, analgesic, and anti-cancer agents. Corresponding studies in the 20th century revealed that *Taraxacum* can be used medicinally, while its inflorescences, leaves, and roots can be processed into different foods. For example, the leaves of cultivated or wild *Taraxacum* species can be eaten in salads, while roots are baked and used as a coffee substitute [5]. Additionally, the *Taraxacum* leaf extract can be used as a flavoring agent for various foods, including alcoholic and soft drinks, frozen dairy desserts, candies, baked goods, pudding, and cheese [6,7].

*The Chinese Pharmacopoeia* (2020 edition) records over forty Chinese patent medicines containing *Taraxacum* genus, which can be clinically utilized for treating over fifty types of diseases. Since *Taraxacum* genus could clear away heat, remove toxicity, disperse swelling, dissipate binds, and induce diuresis, dandelion in clinic is commonly used to cure inflammation, stomach trouble, tumors, gynecological diseases, male urinary system diseases, etc. For example, clinical studies demonstrated that the Kangfuxiaoyan suppository containing dandelion could attenuate symptoms and improve immunity in pelvic inflammatory patients; Rupixiao granule containing dandelion possessed the beneficial activity to induce diuresis so as to mitigate edema and resolve hard lumps, which had a satisfactory effective rate for treating breast hyperplasia. Additionally, according to the ClinicalTrials.gov database resource (https://www.clinicaltrials.gov, accessed on 14 June 2023) supported by the U.S. National Library of Medicine, for the interventional clinical trials of *Taraxacum* genus, one clinical study (NCT00442091) has also been conducted by Odense University Hospital to explore the beneficial effect of dandelion juice on dyshidrotic hand eczema.

Therefore, a comprehensive review of the *Taraxacum* genus studies is necessary considering its numerous benefits. In this work, we reviewed the recent studies of the *Taraxacum* genus. Firstly, we introduced the dandelion herbs and described them both botanically and ethnopharmacologically. We then discussed the main chemical composition of *Taraxacum* genus and outlined its pharmacological effects and toxicity, as these previously have not been fully reviewed to date. Finally, we focused on the challenges and future directions of *Taraxacum* genus, which could elucidate their pharmaceutical research and practical clinical applications. Thus, this review will provide a better understanding of the *Taraxacum* genus and its biological activities by comprehensively summarizing the existing literature.

## 2. Materials and Methods

The literature review was performed using several resources, including Web of Science, ScienceDirect, PubMed, Wiley Online Library, Google Scholar, Europe PMC, Baidu Scholar, American Chemical Society (ACS), and SpringerLink as well as books on Chinese Pharmacopoeia and China Flora, by using different relevant keywords. Additionally, we used Ph.D. and M.S. dissertations, local magazines, and books on toxicology, such as *Ben Cao Gang Mu* and the *Handbook for the Toxicity of Traditional Chinese Medicine*.

The keywords used included *Taraxacum*, dandelion, phytology, genus, ethnopharmacology, sesquiterpenoids, phenolics, other main active components, toxicology, and pharmacological and clinical study. We verified the Latin names of all plants mentioned in this paper from http://www.theplantlist.org/, accessed on 1 January 2023 or http://mpns.kew.org/mpns-portal/, accessed on 1 January 2023 and also provided their validated species names.

## 3. Phytology and Ethnopharmacology

### 3.1. Phytology

Dandelion is widely distributed in the northern hemisphere and belongs to the Asteraceae family. The whole grass possesses medicinal values, such as clearing away heat, detoxification, reduction of swelling, and phlegm dispersion. Generally, the leaves of various dandelions are ≥5–25 cm long, simple, lobed, and form a basal rosette above the central taproot. Their flower heads open during the daytime and close at night, and have colors ranging from yellow to orange. The heads are borne singly on a hollow stem (scape), which is usually leafless and rises 1–10 cm or more above the leaves. Stems and leaves exude white and milky latex when broken. A rosette may simultaneously produce several flowering stems. The flower heads are 2–5 cm in diameter and entirely comprise ray florets. The flower heads mature into spherical seed heads, sometimes called blowballs or clocks, which contain many single-seeded fruits called achenes. Each achene is attached to a pappus with fine hair-like material, which facilitates wind-aided dispersal over long distances (https://en.wikipedia.org/wiki/Taraxacum/, accessed on 1 January 2023 ).

### 3.2. Ethnopharmacology

In traditional Chinese medicine, dandelion was first recorded in *Tang Bencao*—the oldest classical medical book written in the Tang Dynasty (657–659 AD). In this book, dandelion was regarded as a powerful medicine to treat breast swelling and pain. Later, the *Yunnan Materia Medica* written by Ren Lanmao (in the Ming Dynasty) described that dandelion displayed a great detoxification effect and could treat various sores. Additionally, dandelion has also been included in the Ming Dynasty masterpiece *Compendium of Materia Medica* edited by Chinese pharmacologist Li Shizhen. This book describes the detailed functions of dandelion and its wide use in many Chinese medicinal prescriptions.

Currently, dandelion has been recorded in the 2020 edition of the *Pharmacopoeia of the People’s Republic of China*. Thirty-nine prescriptions included dandelion as the principal active component, and these are listed in the *Chinese Pharmacopeia* and approved by the state administration of traditional Chinese medicine (TCM) of the People’s Republic of China (Table 1). Furthermore, many countries also recognize the medical applications of dandelion ever since the advent of ancient medicine, especially in Latin America, Europe, and Asia. Ancient medicine records showed that every part of the dandelion could be used. Based on the symptoms, the usage forms of dandelion may be countless, including infusion, decoction, tincture, plaster, and powder. Furthermore, the administration route may either be oral or topical. The main therapeutic indications include gastrointestinal, skin, and respiratory diseases [1]. Table 2 lists the different uses of the various species of *Taraxacum*.

## 4. Chemical Compounds

Traditional medicinal plants have a corresponding therapeutic effect depending on their constituent compounds [32]. Dandelion is highly regarded for its unique biological characteristics and good biological activity. Considering its excellent pharmacological properties, researchers have isolated their active ingredients over the past few decades. Its biological activity is determined by complex chemical components, mainly sesquiterpenoids, phenolic compounds, essential oils, saccharides, flavonoids, sphingolipids, triterpenoids, sterols, coumarins, etc. In vivo and in vitro studies have displayed outstanding bioactivities of dandelion, such as anti-bacterial, anti-oxidant, anti-cancer, anti-rheumatic, etc. Undoubtedly, it is the diverse phytoconstituents that provide dandelion with remarkable pharmacological properties. For example, phenolic acids such as caffeic acid, coumaric acid, dihydrosyingin, chicoric acid, vanillin, etc., in dandelion possess anti-oxidative and immunostimulant properties. The main sesquiterpene compounds in dandelion are sesquiterpene lactones, usually in the form of glycosides, such as sonchuside, cichorioside C, ixerin D, taraxafolide, and so on, which have anti-inflammatory and anti-bacterial activities. Triterpenoids and sterols in dandelion such as lupenyl acetate, α-amyin acetate, β-amyin acetate, β-sitosterol, daucosterol, etc., can alleviate cardiovascular diseases. Flavonoids in dandelion such as quercetin, chrysoeriol diosmetin, luteolin, etc., usually have anti-oxidative activity; While coumarins in dandelion such as aesculin, cichoriin, esculetin, scopoletin, etc., possess anti-inflammatory, bacteriostatic, anti-coagulant, and anti-cancer effects.

### 4.1. Sesquiterpenoids and Phenolic Compounds

**Figure 2:**
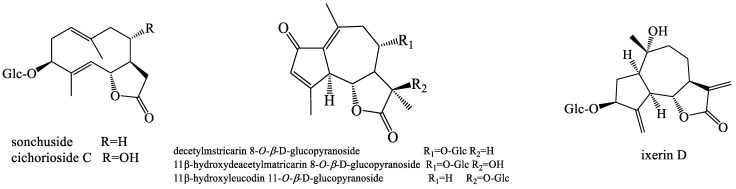

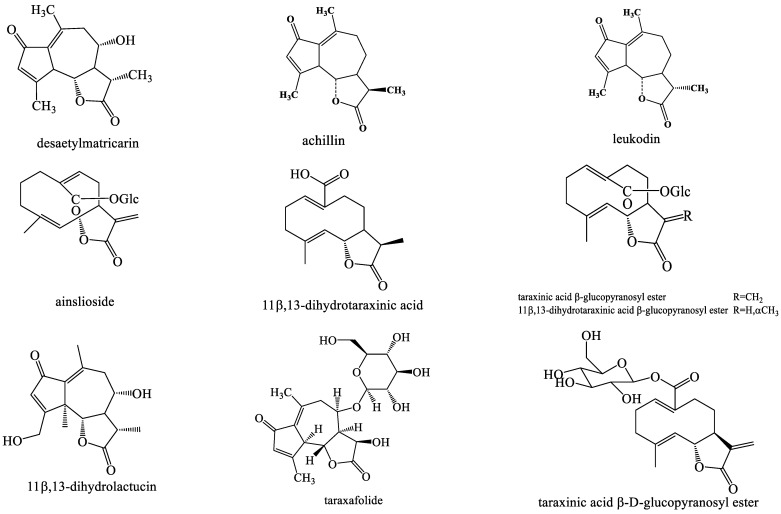
The chemical structures of representative sesquiterpenoids from *Taraxacum* genus.

Dandelion contains different sesquiterpenoid and phenolic compounds. The bitter taste of dandelion is mainly imparted by sesquiterpenoids (Figure 2). The only known sesquiterpene lactone components in this plant are two germacranolides, namely, taraxinic acid and the β-glucopyranosyl ester and its 11, 13-dihydroderivative and two eudesmanolides (4a(15),11β(13)-tetrahydroridentin B, and taraxacolide-1-*O*-β-glucopyranoside), which were isolated from *T. officinale* [33]. From the ethanolic root extracts of two different species of the *Taraxacum* genus (*T. laevigatum and T. disseminatum*), eight types of germacrane and eudesmane sesquiterpenoids, including 1β,3β,6α-trihydroxy-4α(15)-dihydrocosticacid methyl ester and its 1-*O*-β-glucopyranoside, were obtained [34]. A sesquiterpenoid ketolactone and a new guaianolide were isolated from an ethyl acetate-soluble part of a methanolic extract of *T. wallichii* [35]. Furthermore, eight sesquiterpenes, including 1β,3β-dihydroxy-eudesman-11(13)-en-6α,12-olide, 1β,3β-dihydroxyeudesman-6α,12-olide, loliolide, 1β,3β-dihydroxyeudesman-11(13)-en-6α,12-olide, ainslioside 1β,3β-dihydroxyeudesman-6α,12-olide, and 11β,13-dihydrotaraxinic acid were successfully obtained from *T. mongolicum* [36,37,38]. Five germacrane- and guaiane-type sesquiterpene lactones together with benzyl glucoside, dihydroconiferin, syringing, and dihydrosyringin were isolated from the roots of *T. officinale* [39]. Eleven sesquiterpene lactones, including the new guaianolide 11β-hydroxydeacetylmatricarin-8-*O*-β-glucopyranoside and four other known phenolic glucosides, were also isolated from the roots of *T. hondoense* [40]. *T. erythrospermum*, *T. serotinum*, *T. obovatum*, *T. alpinum*, and *T. udum* are species of the *Taraxacum* genus. Additionally, sixteen sesquiterpenoids were also isolated from the aforementioned varieties [41,42,43,44,45]. Two eudesmane-type sesquiterpene lactones, (2β-hydroxysantamarine-1β-D-glucopyranoside and 3β-hydroxy-4α*H*-3-dihydrosantamarine-β-d-glucopyranoside) were successfully isolated from the methanolic extract of *T. linearisquameum* [46]. New sesquiterpenoids were also obtained from the roots of *T. platycarpum* [47]. In 1998, a guaianolide sesquiterpene, desacetylmatricarin, was isolated from *T. platycarpum* and reported as an active ingredient with the anti-allergic property [48]. 14-*O*-β-d-Glucosyl-l1,13-dihydro-taraxinic acid and 14-*O*-β-d-glucosyl-taraxinic acid were extracted from the roots of *T. officinale* [49].

**Figure 3:**
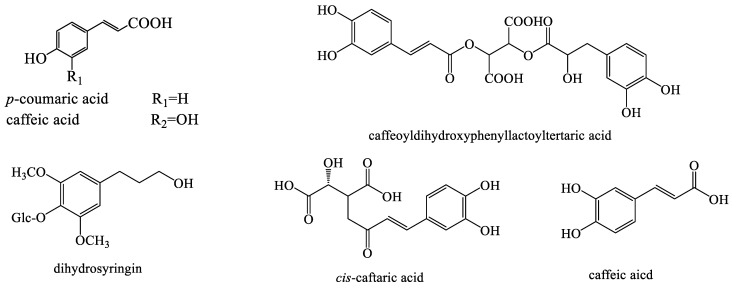

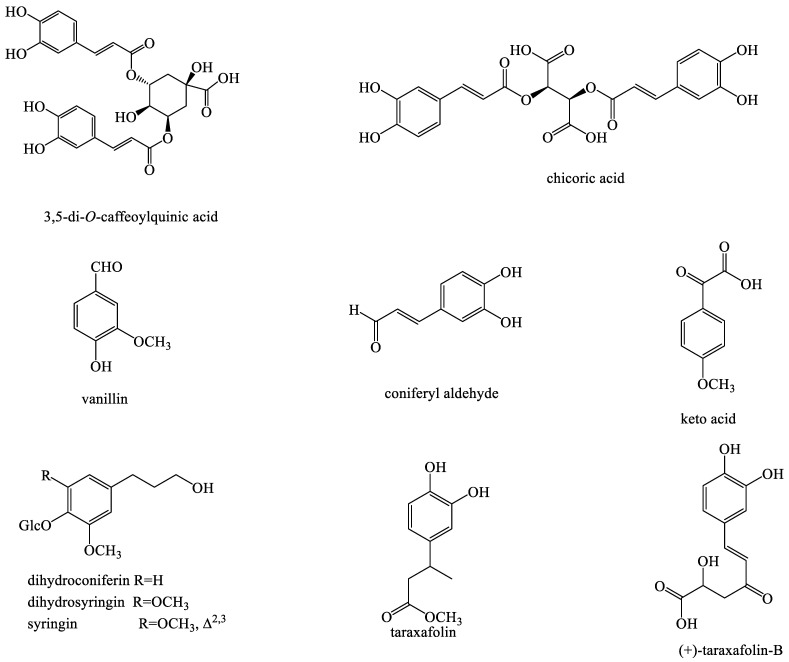
The chemical structures of representative phenolic compounds from *Taraxacum* genus.

Polyphenolic compounds are widely present in plants. Many potentially active phenolic compounds are isolated from different species of the *Taraxacum* genus [50,51,52] (Figure 3).

### 4.2. Essential Oils

Bylka et al. analyzed *T. officinale* L. by gas chromatography–mass spectrometry (GC–MS) and obtained 25 volatile compounds, with 1,3-dimethylbenzene, 1,2-dimethylbenzene, 1-ethyl-3-methylbenzene, heneicosane, and tricosane as the main components [53]. The separated compounds also included straight-chain aliphatic hydrocarbons (nonadecane, hexadecane, heneicosane, pentadecane, tricosane, eicosane, and 1-tridecyne), branched aliphatic hydrocarbons (2,5,5-trimethylheptane and 6-ethyl-2-methyloctane), esters (benzyl benzoate), alkylated benzenes (1,3-dimethylbenzene, 1,2-dimethylbenzene, 1-ethyl-3-methylbenzene, and 1-hydroxymethyl-4-methylbenzene), alcohols (2-nonen-1-ol, 1,9-nonanediol, and 1-tridecanol), aldehydes (octanal, phenylacetaldehyde, 2-methylbenzaldehyde, nonanal, pentadecanal, and 10-undecenal), and ketones (5-methyl-2-hexanone and hexadecanoic acid). Upon analyzing the volatile components of *T. officinale* using GC–MS, the essential oil components obtained were butyl acetate, 2-methyl-propanol, *n*-butanol, 4-phenyl-1-butanol, 4-hydroxyl 4-methyl-2-pentanone, acetic acid, 4-terpineol, fluoro-terpineol, and alpha-terpineol [54].

### 4.3. Saccharides

A recent study reported the successful extraction of a water-soluble heteropolysaccharide from *T. mongolicum Hand.-Mazz* comprising three monosaccharides, namely pika, arabinose, and galactose in a molar ratio of 1.0:10.7:11.9 [55]. Schütz et al. isolated fructooligosaccharides and fructopolysaccharides from the root of *T. officinale WEB.* ex WIGG [56].

### 4.4. Flavonoids

**Figure 4:**
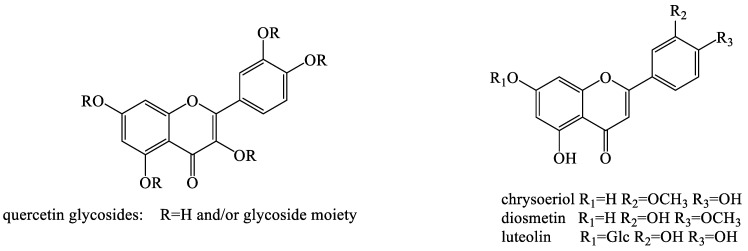

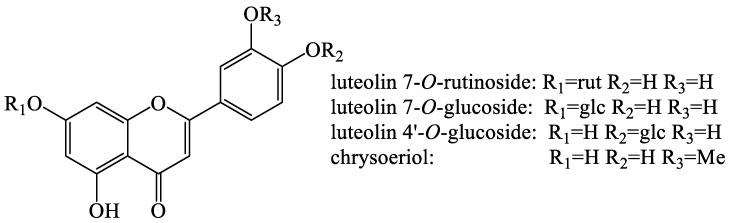
The chemical structures of representative flavonoids from *Taraxacum* genus.

Flavonoids are a class of natural compounds with a 2-phenylchromanthone structure and a ketone carbonyl group. The oxygen atom in the first position is basic and can form a salt with a strong acid. The hydroxy derivative has a yellow color and is called a xanthophyll or a flavonoid. Dandelion contains diverse flavonoids, which are important in plant growth, development, flowering, fruiting, and anti-bacterial defense (Figure 4). Six flavonoids, including apigenin, luteolin, quercetin, luteolin-7-β-d-glucopyranoside, quercetin-7-β-d-glucopyranoside, and quercetin-37-*O*-β-d-diglucopyranoside, were obtained and identified from *T. mongolicum* [57]. Two new flavone glycosides, namely, isoetin-7-*O*-β-d-glucopyranosyl-2′*-O*-α-L-arabinopyranoside and isoetin-7-*O*-β-d-glucopyranosyl-2′-*O*-α-d-glucopyranoside, were isolated from the aerial part of *T. mongolicum*. The structures of these compounds were elucidated mainly by spectral analyses [58]. Shi et al. established an online rapid screening method, namely, high-performance liquid chromatography (HPLC) diode array for detection and electrospray mass spectrometry system for separation and identification of free radical scavengers in *T. mongolicum* methanol extract. Additionally, the detected anti-oxidant was directly separated by preparative HPLC (PHPLC) and Sephadex LH-20. The purified compound was sampled using an off-line nuclear magnetic resonance (NMR) spectrometer to obtain the corresponding spectrum. Thirty-two kinds of free radical scavenging compounds were screened, isolated, and identified, including 16 flavonoids, 10 phenylpropyl compounds, and 6 benzoic acid compounds. Among them, 17 compounds were isolated for the first time from *T. mongolicum*, including three new compounds [59]. Five flavonoid glycosides were isolated and purified from the gas phase of *T. mongolicum* (a traditional Chinese medicinal herb) using high-speed counter-current chromatography (HSCCC) [60,61,62]. Moreover, two polymethoxylated flavones were isolated from *T. mongolicum* in 2009 [63]. In 1996, three flavonoid glycosides, including luteolin 7-glycoside and two luteolin 7-diglucosides, were isolated from the flowers and leaves, while free luteolin was isolated from the flower tissues of *T. officinale* [63]. Eight flavones and eight flavonol glycosides were isolated from *T. officinale* WEB. ex WIGG. and identified using HPLC/electrospray ionization mass spectrometry [50]. Ten flavonoids were identified from *T. formosanum* and quantified with concentrations of 9.9–325.8 μg g^−1^ [51]. 

### 4.5. Sphingolipids

**Figure 5:**
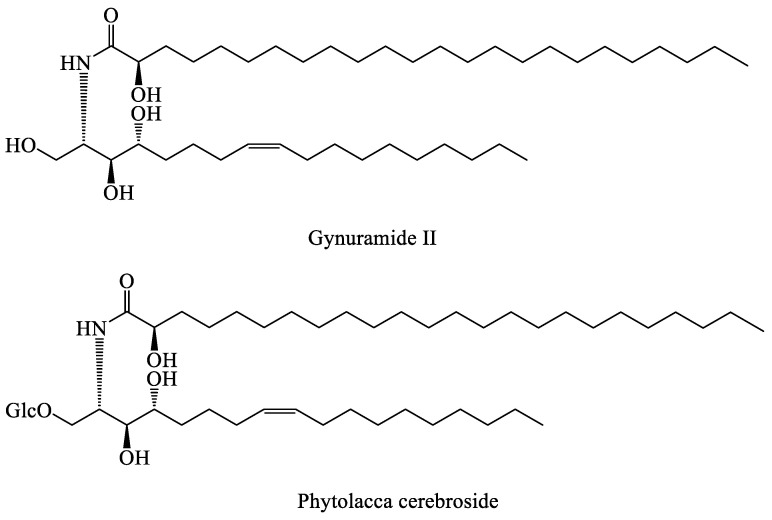
The chemical structures of representative sphingolipids from *Taraxacum* genus.

A sphingolipid comprises a long-chain fatty acid, a sphingosine molecule or its derivative, and a polar head alcohol. The polar head group of the sphingolipid binds to the hydroxyl group of the sphingosine, while the fatty acid moiety forms an amide bond with its amino group. Two sphingolipids, namely, gynuramide II and phytolacca cerebroside, were obtained and identified from the root of *T. mongolicum* [57] (Figure 5).

### 4.6. Triterpenoids and Sterols

**Figure 6:**
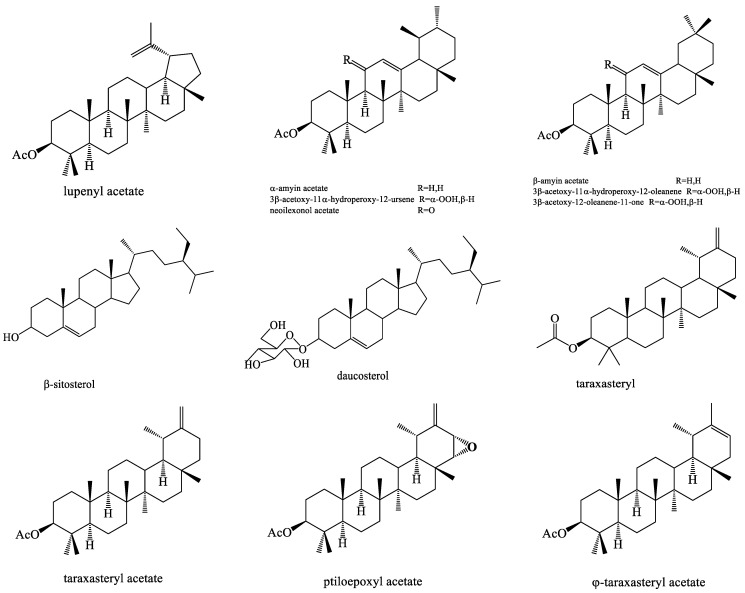
The chemical structures of representative triterpenoids and sterols from *Taraxacum* genus.

Triterpenoids are substances formed by the end-to-end joining of several isoprenes with their hydroxyl groups being removed. Most triterpenoids comprise a chain of 30 carbon atoms, while few contain 27 carbon atoms. In dandelion, pentacyclic triterpenoids are the main type. A sterol, which is a general term for a group of compounds with a fluorene nucleus, has a cyclopentane polyhydrophenanthrene skeleton. There are different kinds of sterols in different parts of the dandelion. In dandelions, triterpenoids and sterols exhibit remarkable anti-oxidative and anti-inflammatory activities (Figure 6). Six triterpenoids and sterols, such as gigantursenol A, taraxasterol, β-sitosterol, β-sitosterol-3-*O*-β-d-glucoside, stigmasterol, and β-sigmasterol-3-*O*-β-d-glucoside were successfully obtained from the root of *T. mongolicum* [57]. Warashina et al. extracted eight new triterpenes from dandelion roots [47]. Later, three novel triterpenoids, including the lupane-, bauerane-, and euphane-type triterpenoids were isolated from the roots of *T. officinale* [64].

### 4.7. Coumarins

**Figure 7:**
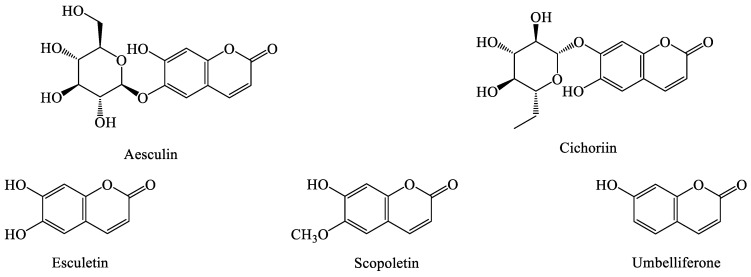
The chemical structures of representative coumarins from *Taraxacum* genus.

Coumarin-based compounds are relatively less distributed in dandelions (Figure 7); 6,7-dihydroxycoumarin (escin) and scutellarin were isolated from the stems and leaves of *T. officinale* in 1981 [65]. Nine coumarin compounds (including umbelliferone, coumestrol, lactucin, hachibate, east azlactone, resveratrol, lactucin, chicory, and esculin) were isolated from *T. officinale* and *T. mongolicum Hand.-Mazz* [30,63,66].

### 4.8. Others

**Figure 8:**
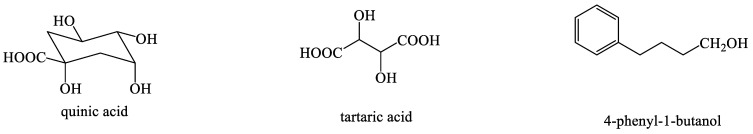

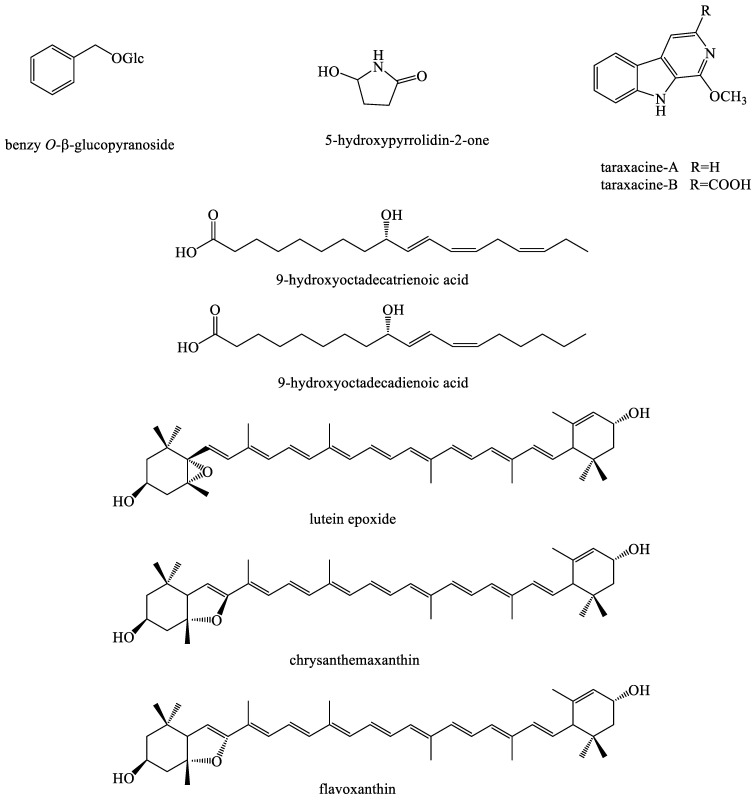
The chemical structures of other types of compounds from *Taraxacum* genus.

Some studies have reported that *T. mongolicum* also contains glycerin, inositol, polysaccharides, and other compounds that are indispensable for plant growth [67,68] (Figure 8). With the advancement of identification techniques, different classes of compounds can be assigned by comparing them with standards. Ma et al. employed ultra-performance liquid chromatography (UPLC) and identified three compounds, namely, chlorogenic acid, caffeic acid, and taraxasterol [69]. Oh et al. used HPLC to identify quercetin from the ethanolic extract of *T. mongolicum* [70]. Lignans, including mongolicumin A and rufescidride, were obtained from *T. mongolicum* [38,60]. The leaf extract of *T. officinale* contained houttuyin and aescin. This study is the first to report the discovery of free pterin (luteolin 3′-methyl ether). The contents of cichoric acid, chlorogenic acid, houttuyin, and aescin in chicory plants were initially identified. Chicoric acid and its related monocaffeine tartaric acid are major phenolics, which are also used in pharmaceutical preparations and found in alfalfa [63]. Jia et al. isolated two compounds, namely, caffeic acid and luteolin 7-*O*-β-d-glucopyranoside, from *T. mongolicum* [71]. HPLC was used to identify an organic acid from *T. mongolicum Hand.-Mazz*; the acid plays an important role in the treatment of acute tracheobronchitis and has good anti-inflammatory activity [72,73]. Kao utilized HPLC–MS spectrometry and carotenoid column chromatography and successfully separated 25 carotenoids from *T. formosanum*. Furthermore, all-*trans*-canthaxanthin was found to be an appropriate internal standard for quantitation; all-*trans*-carotene and its cis isomers had the largest amount (413.6 μg·g^−1^), followed by all-*trans*-violoxanthin and its cis isomers (209.5 μg·g^−1^), all-*trans*-lutein and its cis isomers (212.4 μg·g^−1^), all-*trans*-neoxanthin and its *cis* isomers (134.6 μg·g^−1^), antheraxanthin (16.5 μg·g^−1^), all-*trans*-cryptoxanthin and its *cis* isomers (5.8 μg·g^−1^), all-*trans*-zeaxanthin (3.6 μg·g^−1^), and neochrome (0.1 μg·g^−1^). Two inositol derivatives, namely, (1S,2S,4R,5S)-2,3,4,6-tetrahydroxy-5-[2-(4-hydroxyphenyl)acetyl] oxycyclohexyl-2-(4-hydroxyphenyl) acetate and (2S,3R,5R,6S)-2,3,5,6-tetrahydroxy-4-[2-(4-hydroxyphenyl) acetyl]oxycyclohexyl-2-(4-hydroxy- phenyl) acetate, were successfully isolated from the methanolic extract of *T. linearisquameum* [74]. Additionally, the known compound taraxinic acid β-D-glucopyranosyl ester was isolated [46]. Kenny et al. used liquid chromatography–mass spectrometry to separate and identify different 4-hydroxyphenylacetic acid derivatives, including, 9-hydroxyoctadecatrienoic acid and 9-hydroxyoctadecadienoic acid, from the root fraction of *T. officinale* and characterized these compounds using spectroscopy [75,76]. Lutein epoxide was successfully isolated from the petals of *T. officinale* F. Weber ex Wiggers. Moreover, all-E-lutein epoxide was the major carotenoid and had high amounts of (9Z)- and (9′Z)-isomers [77]. Phytochemical investigation of the roots of *T. coreanum* led to the isolation of two new inositol derivatives [52].

## 5. Pharmacological Effects

**Figure 9:**
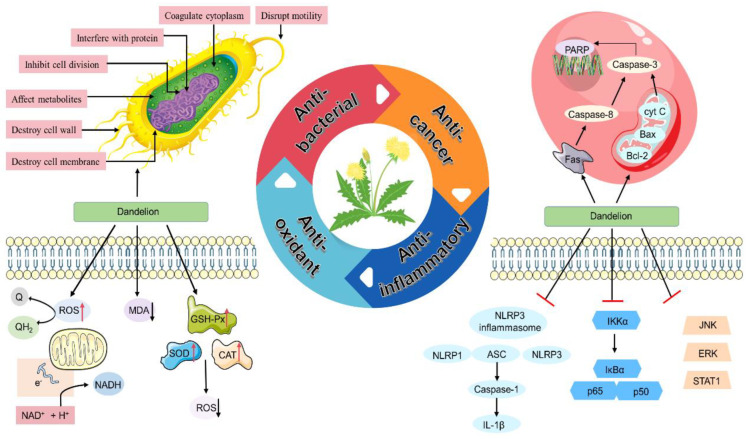
The pharmacological mechanisms of the key chemical constituents of the dandelion.

Dandelion has been reported to have multiple pharmacological effects, including anti-bacterial, anti-oxidant, anti-cancer, anti-rheumatic, etc. This section reviews recent findings on the pharmacological effects of *Taraxacum* (Figure 9) (Table 3).

### 5.1. Anti-Bacterial and Anti-Oxidant Effects

Dandelion reportedly possesses excellent anti-bacterial activity. Díaz and his colleagues isolated diverse chemical compounds from *T. officinale* leaves, mainly triterpenoids and other unknown compounds. Subsequently, the leaves’ extract could markedly inhibit Gram-positive bacteria with a minimum inhibitory concentration (MIC) of 200 g mL^−1^), thus suggesting dandelion had promising anti-bacterial potential [127]. In another experiment, the content, anti-oxidant activity, and cytotoxicity of phenols and flavonoids in three different types of dandelion methanolic extracts were investigated. The total phenolic content was 1000 mg·kg^−1^, with the aboveground content being higher than the root. *T. mongolicum* had the highest phenolic content in the stems (76.8 mg·kg^−1^) and roots (40.0 mg·kg^−1^), followed by *T. coreanum* and *T. officinale* (*p* < 0.05). Furthermore, the total flavonoid content also showed a consistent trend with the total phenolic content. The anti-oxidant activity of each methanolic extract increased in a dose-dependent manner. The maximum 2,2-diphenyl-1-picrylhydrazyl (DPPH) free radical scavenging activities of *T. mongolicum* shoot and root extracts (89.6% and 83.4%) were obtained at a concentration of 1000 mg·kg^−1^. The overall experimental results proved that the total phenolic and flavonoid levels were highly correlated with anti-oxidant activity, but their content and activity varied across species [87,89]. In another study of the anti-bacterial activity of the extracts of *T. mongolicum* in different solvents, Gao reported that only the ethanolic extract had varying degrees of anti-bacterial activity (inhibition zone >7 mm in diameter), while the aqueous extract did not [78].

Flavonoids and coumaric acid derivatives were extracted from dandelion flowers. In the study of anti-oxidant properties, the extracts had scavenged effects on superoxide and hydroxyl radical-induced damage; meanwhile, the inhibition of hydroxyl radicals was nonspecific. The reduction in the phenolic content of the extract reduced the DPPH capacity and showed a synergistic effect with α-tocopherol. After the addition of the corresponding extract, the bacterial lipopolysaccharide (LPS) stimulated macrophage RAW264.7 cells in mice, thereby significantly reducing the NO concentration in a concentration-dependent manner. Additionally, adding a certain extract concentration significantly inhibited the peroxide radical-induced intracellular oxidation of RAW264.7 cells. The extract demonstrated a significant anti-oxidant activity in biological and chemical models. In addition, the inhibitory effect of the extract on reactive oxygen species (ROS) and NO was related to its phenolic content [93].

*T. mongolicum* extract inhibited four Gram-negative bacteria and two Gram-positive bacteria, especially *Pseudomonas aeruginosa* and *Bacillus subtilis*, with MIC values of 125 and 62.5 μg·mL^−1^, respectively. The ethyl acetate soluble component extracted from dandelion had high anti-bacterial activity and can be used as a natural preservative in the pharmaceutical industry [79]. The relationship between osteoporosis and oxidative stress induced by ROS was also studied and food and plants with anti-oxidant effects are being increasingly focused upon to reduce the ROS-induced damage caused during bone metabolism. The anti-oxidative effect of *T. mongolicum* on the proliferation and the differentiation of MC3T3-E1 cells induced by hydrogen peroxide was investigated, and the total contents of polyphenols and flavonoids were 33.65 and 4.45 mg·g^−1^, respectively. Under hydrogen peroxide-induced oxidative stress, dandelion extract promoted the proliferation of MC3T3-E1 cells and differentiation of osteoblasts. Hence, dandelion extract can inhibit oxidative stress-induced damage to osteoblasts and serve as a potential anti-oxidant material for preventing bone diseases [94].

With the increasing resistance of cow mastitis to bacteria and considering the safety of dairy products, anti-bacterial extracts should be used instead of antibiotics for the treatment of mastitis in dairy cows. The anti-bacterial effects of purslane and *T. mongolicum* aqueous and ethanolic extracts on the main pathogens of cow mastitis (*Escherichia coli*, *Staphylococcus aureus*, *Streptococcus agalactiae*, and *Streptococcus agalactiae*) were studied using disk diffusion method. The aqueous and ethanolic extracts of the two traditional Chinese medicines had different inhibitory effects on the four pathogens of cow mastitis. The anti-bacterial activity of the two Chinese herbal extracts against *E. coli* was higher than that against other bacteria. The ethanolic extract had higher anti-bacterial activity against *E. coli* than purslane. However, the anti-bacterial activity of the *T. mongolicum* ethanol extract was lower than that of the aqueous extract. Hence, purslane and the *T. mongolicum* extract may be used for the treatment of mastitis in dairy cows [66].

### 5.2. Anti-Cancer Effects

A previous report investigating the use of *T. mongolicum* extracts for the prevention and treatment of bovine mastitis discovered that different concentrations of the extract had no noticeable cytotoxic effect on MAC-T cells, thus being the first to report that the *T. mongolicum* extract significantly inhibited the production of NO and pro-inflammatory cytokines in MAC-T cells. This finding has a certain clinical application value for the prevention and treatment of bovine mastitis [108]. Breast cancer is an aggressive and fatal breast disease with limited treatment options. Although *T. mongolicum* (a Chinese herbal medicine with anti-cancer activity) has been used for the treatment of breast abscess and breast hyperplasia since ancient times, its mechanism of action needs further scientific studies [128]. *T. mongolicum* extract significantly inhibited the activity of MDA-MB-231 cells by causing the G2/M phase arrest and apoptosis. The extract also significantly increased the levels of cleaved caspase-3 and PARP proteins, with the caspase inhibitor Z-VAD-FMK inhibiting *T. mongolicum* extract-induced apoptosis. Three ER stress-related signals were strongly induced by the *T. mongolicum* treatment, including increased expression of *ATF4*, *ATF6*, *XBP1s*, *GRP78*, and cleavage-related genes along with elevated phosphorylation levels of proteins, eIF-2αIRE1, and downstream molecular GRP78 impermanence. MDA-MB-231 cells transfected with CHOP siRNA significantly inhibited the *T. mongolicum* extract-induced apoptosis. The underlying mechanism is partially attributed to the strong activation of the active/p-eIF2α/ATF4/cut axis. In conclusion, apoptosis induced by endoplasmic reticulum stress generates the anti-cancer effect of the *T. mongolicum* extract, thereby suggesting that the *T. mongolicum* extract may be a potential treatment for triple-negative breast cancer (TNBC) [57]. However, the use of dandelion for breast cancer treatment is mainly based on anecdotal evidence and has no sufficient scientific evidence. Therefore, Oh et al. hypothesized that *T. mongolicum* can act as a selective estrogenic receptor modulator and hormone replacement therapy for postmenopausal women. *T. mongolicum* ethanol extract significantly increased the cell proliferation and estrogenic response element-driven luciferase activity. Hence, *T. mongolicum* ethanol extract can induce estrogenic activity mediated by the classical estrogenic receptor pathway, thereby providing a scientific basis for its anti-cancer application in traditional medicine [70].

### 5.3. Anti-Inflammatory Effects

Inflammation plays an important role in the pathogenesis of acute tracheobronchitis. The main component of *T. mongolicum Hand.-Mazz*—an organic acid, has good anti-inflammatory activity. Furthermore, organic acids can improve the regulation of the TLR4/NF-κB (TLR4/IKK/NF-κB) signaling pathway in LPS-mediated histopathological damage, which may provide a basis for the treatment of acute tracheobronchitis [72,73].

*T. mongolicum* is widely used in the Eastern Hemisphere. Since *T. mongolicum* has a high mineral content, it causes potential problems with the absorption of quinolones. Since a previous study reported the occurrence of multifactorial drug interactions between *T. mongolicum* and ciprofloxacin, the effects of their simultaneous use should not be ignored. Ciprofloxacin is a fluoroquinolone antibiotic that has good anti-bacterial activity against Gram-positive, Gram-negative, and mycobacteria. However, its oral absorption greatly diminished the effect of simultaneous administration of metal-containing cations. This phenomenon has been extensively studied for antacids, mineral supplements, and dairy products. However, information about this interaction is not yet available in mineral-rich herbal and health foods. Drug–drug interactions may occur between ciprofloxacin and a mineral-rich anti-inflammatory/anti-bacterial herb, *T. mongolicum Hand-Mazz.* Traditionally, *T. mongolian* dried plants are used for the treatment of lice, ulcers, mastitis, lymphadenitis, inflamed eyes, sore throat, lung and breast abscesses, acute appendicitis, jaundice, and urinary tract infections. Furthermore, this herb exerts a bactericidal effect on multiple pathogens, and its water extract has MIC values ranging from 1:10 to 1:640. In addition, the in vitro antifungal, anti-leptospiral, and antiviral effects of the herbs have been proven. Chemical testing of *T. mongolicum* indicates the presence of triterpenoids (such as tartaric alcohol and tartaric acid), inulin, pectin, asparagine, and phenolic compounds. A comprehensive pharmacokinetic assessment of the rat was performed and demonstrated the potential of drug–drug interactions between *T. mongolicum* and ciprofloxacin [80].

Different solvent extracts of *T. officinale* were successfully prepared by Jeon et al. in a carrageenan-induced balloon model [104]. The ethanolic extract inhibited the production of exudates and significantly reduced the NO and leukocyte levels in the exudate. The extract also inhibited acetic acid-induced vascular permeability in a dose-dependent manner in acetic acid-induced abdominal peristalsis in mice. In summary, medicinal dandelion has anti-angiogenic, anti-inflammatory, and anti-nociceptive properties by inhibiting NO production and cyclooxygenase-2 (COX-2) expression and/or its anti-oxidant activity.

Mouse macrophages (RAW 264.7) were used to study the anti-inflammatory effects and mechanism of the methanolic extract of *T. officinale* leaves on LPS induction. The methanolic extract and its components inhibited LPS-induced production of NO, pro-inflammatory cytokines, and prostaglandin (PG) E_2_ in a dose-dependent manner. However, the chloroform soluble fraction significantly inhibited the production of NO, PG E_2_, and two pro-inflammatory cytokines (tumor necrosis factor-α (TNF-α) and interleukin-1β (IL-1β)) in a dose-dependent manner, with MIC values of 66.51, 90.96, 114.76, and 171.06 μg·mL^−1^, respectively. Hence, the anti-inflammatory effects of leaf extract may be due to the down-regulation of NO, PG, E_2_, and pro-inflammatory cytokines along with the inactivation of the MAP kinase signaling pathway, thereby reducing the expression of inducible NO synthase (iNOS) and COX-2 [107]. Therefore, in the anti-inflammatory mechanism study, the aqueous extract of *T. mongolicum* exerted certain protective effects on acute lung injury-induced inflammation in mice [69].

## 6. Other Effects

Baek examined the methanolic extract of *T. mongolicum* and its fraction for their scavenging effects on DPPH and superoxide radicals and also their hepatoprotective effects on tacrine-induced cytotoxicity in the human hepatoma cell line, HepG2 cells. The extract had free radical scavenging and hepatoprotective effects [129]. The novel homogenous polysaccharide DPSW-A was obtained from *T. mongolicum* and its derivative demonstrated limited anti-coagulant function [55]. Furthermore, the newly isolated compound 1β,3β-dihydroxy-eudesman-11(13)-en-6α from *T. mongolicum* inhibited the NO production, with an IC_50_ of 38.9 μM [36].

In the study of its hypolipidemic action and mechanism, *T. mongolicum* was extracted separately with water, 50% ethanol, and 95% ethanol. The 50% ethanolic extract was the most effective among the 13 extracts. Prolonged administration of the 50% ethanolic extract significantly reduced the body weight of rats and the serum levels of triglyceride LDL-C and total cholesterol. Hence, *T. mongolicum* helps in lowering blood lipid levels [68]. Moreover, the *T. mongolicum* methanol extract strongly inhibited monoamine oxidase. Therefore, the extract can potentially affect diseases, such as depression, dementia, and Alzheimer’s disease [95].

Skin whitening is becoming popular among people. Melanin is an important factor that determines skin color. In the study of melanin synthesis inhibition by *T. mongolicum* extract, reverse-transcriptase polymerase chain reaction and Western blot were used to analyze the protein and mRNA levels of tyrosinase-related protein (TRP)-1, TRP-2, tyrosinase, MITF, ERK, and PKA, and it was found to inhibit melanin synthesis [110].

To verify the antiviral effect of *T. mongolicum* on the hepatitis B virus, researchers found that 50–100 g·mL^−1^ *T. mongolicum* extract could protect the rat hepatocytes as compared with D-galactosamine (D-GalN), thioacetamide (TAA), and t-butyl hydroperoxide (t-BHP). The protective effect of 100 g·mL^−1^ *T. mongolicum* extract on rat hepatocytes was enhanced. Furthermore, the *T. mongolicum* extract significantly inhibited DNA replication at 1–100 g·mL^−1^, and reduced the levels of HBsAg and HBeAg at 25–100 g·mL^−1^, with inhibition rates of 91.39% and 91.72% at 100 g·mL^−1^, respectively. The *T. mongolicum* extract significantly inhibited DNA replication at 25–100 g·mL^−1^, thus exerting a strong antiviral effect on HBV. The protective effect of *T. mongolicum* extract on hepatocytes may be achieved by inhibiting oxidative stress. However, the antiviral properties of the *T. mongolicum* extract may help block protein synthesis and DNA replication. The main components of the *T. mongolicum* extract were quantitatively analyzed to provide a scientific basis for its use in the treatment of hepatitis [71].

## 7. Toxicity

When a plant or a compound isolated from a plant has no significant toxicity or side effects, its potential therapeutic effect should be studied further. This is particularly important for dandelion [130]. In daily life, the recommended dosage of dandelion is 10–15 g [1]. In 1974, Râcz–Kotilla et al. [114] studied the diuretic effect of a 4% aqueous extract of dandelion. Firstly, they performed acute toxicity tests on different parts of dandelion and fluid extracts of grass (DL_50_ = 27.2 g·kg^−1^ body weight). In the diuretic experiment, the aqueous extract of dandelion was administered at a dose of 8 g·kg^−1^ body weight for one month, and the body weight of the mice and rats were found to be reduced by ~30%. In the study of the protective effect of renal oxidative damage caused by CCl_4_, the oral administration of 100, 250, 300, 500, and 750 mg·kg^−1^ dandelion aqueous extract for the duration of the test was considered safe. In addition, the corresponding lesions in mice showed a good prognosis, thus indicating that these dosages are within the normal range [115,122,131]. In terms of cytotoxicity, HepG2, HeLa, HL60, and Vero E6 cells had different IC_50_ values (0.015 ± 0.001, 0.023 ± 0.002, >0.25) [92].

## 8. Conclusions and Future Prospects

As a well-known complementary and alternative medicine, the whole dandelion herb, including its roots, stem, leaf, flower, and seed is rich in diverse bioactive ingredients including sesquiterpenes, phenolic compounds, phytosterols, triterpenes, etc. However, previous studies have mainly focused on extracting and identifying active ingredient structures from different kinds of dandelion. At present, most research focuses on studying the biological activity of partial extracts such as the root extracts, while the research on the biological activity of other effective active ingredients of dandelion are relatively fewer. Moreover, the pharmacological research of the effective active ingredients of dandelion mostly focuses on the basic pharmacological mechanism, and the form of mechanism research is relatively simple. For example, in order to clarify the specific anti-cancer mechanism of dandelion, more advanced strategies including network pharmacology, molecular pharmacology, and metabolomics methods can be flexibly used to comprehensively demonstrate the multi-target anti-cancer action mechanism of dandelion, which will provide new insights for further accurate search, and confirmation and optimization of the relationship between the active ingredient of dandelion and the target. Additionally, most of the current studies focus on in vitro cell experiments, and the research results lack clinical applicability. In the future, a large number of in vivo animal models are needed to deeply study the pharmacological mechanisms and targets of active ingredients of dandelion, so that they can realize clinical application as soon as possible and offer new ideas and methods for precise treatment.

To be more specific, *T. mongolicum*, *T. borealisinense*, *T. coreanum Nakai*, and *T. officinale* are the most frequently utilized species for complementary and alternative medicine. Additionally, in terms of pharmacological effects, dandelion could exert potent anti-bacterial, anti-oxidant, anti-cancer, and anti-rheumatic activities. Moreover, the extracts from different parts all displayed excellent aforementioned activities, which provided strong evidence regarding the use of the traditional medicinal herb as an anti-bacterial drug. However, the anti-bacterial effect differed between the different types of dandelion. Although dandelion is a traditional medicinal plant used for different treatments, its mechanism of action and its corresponding biological activity and safety should be further studied. Furthermore, when dandelion is clinically applied, in-depth research and investigation should be conducted regarding its distribution and metabolism. Therefore, we believe that with further developments in science and technology, novel drug technologies can be combined with traditional therapeutic medicinal plants, such as dandelion, to achieve better treatment outcomes.

## Figures and Tables

**Table 1:** Preparations in which *Taraxacum mongolicum* Hand.-Mazz. is the main component listed in *Chinese Pharmacopoeia* and approved by the government.

Preparation Name	Type	Main Compositions	Function	Administration	Storage
Yiganning Keli	Granules	*Hedyotis diffusa*; *Artemisia capillaris* Thunb.; *Lysimachia christinae* Hance; *Codonopsis pilosula* (Franch.) Nannf.; *T. mongolicum* Hand.-Mazz.; *Reynoutria multiflora* (Thunb.) Moldenke; *Paeonia suffruticosa* Andr.; *Smilax glabra* Roxb.; *Paeonia lactiflora* Pall.; *Melia toosendan* Sieb. et Zucc.	Invigorating the qi and strengthening the spleen, activating blood and removing phlegm, removing heat, detoxification	Orally	Sealed storage
Erding Keli	Granules	*Viola yedoensis* Makino; *Lobelia chinensis* Lour.; *T. mongolicum* Hand.-Mazz.; *Strobilanthes cusia*	Detoxification	Orally	Sealed storage
Xiao’er Baotaikang Keli	Granules	*Forsythia suspensa* (Thunb.) Vahl; *Rehmannia glutinosa* (Gaertn.) Libosch. ex Fisch. & C.A. Mey.; *Bupleurum chinense* DC.; *Scrophularia ningpoensis* Hemsl.; *Fritillaria thunbergii* Miq.; *T. mongolicum* Hand.-Mazz; *Strobilanthes cusia*; *Arnebia euchroma* (Royle) Johnst.	Clearing the heat, relieving cough and phlegm	Orally	Sealed storage
Xiao’er Jiebiao Keli	Granules	*Lonicera japonica* Thunb.; *Forsythia suspensa* (Thunb.) Vahl; *Arctium lappa* L.; *T. mongolicum* Hand.-Mazz; *Scutellaria baicalensis* Georgi; *Saposhnikovia divaricata* (Turcz.) Schischk.; *Perilla frutescens*; *Schizonepeta tenuifolia* Briq.; *Pueraria lobata* (Willd.) Ohwi; Cow-bezoar	Diffusing the lungs, clearing heat, detoxification	Orally	Sealed storage
Niuhuang Jingnao Pian	Troche	Cow-bezoar; *Lonicera japonica* Thunb.; *Forsythia suspensa* (Thunb.) Vahl; *Scutellaria baicalensis* Georgi; *Coptis chinensis* Franch.; *T. mongolicum* Hand.-Mazz; Pearl; *Ophiopogon japonicus* (Thunb.) Rehmannia glutinosa (Gaertn.) Libosch. ex Fisch. & C.A. Mey.	Clearing away heat, detoxification	Orally	Sealed and protected from moisture
Shuanghu Qinggan Keli	Granules	*Lonicera japonica* Thunb.; *T. mongolicum* Hand.-Mazz; *Chrysanthemum indicum* L.; *Bunge Salvia miltiorrhiza* Bunge; *Reynoutria japonica* Houtt.; *Coptis chinensis* Franch.	Clearing away heat and dampness, dispelling phlegm and widening the qi, promoting qi and promoting blood circulation	Orally	Sealed storage
Baipuhuang Pian	Troche	*Pulsatilla chinensis* (Bge.) Regel; *T. mongolicum* Hand.-Mazz; *Scutellaria baicalensis* Georgi; *Phellodendron amurense* Rupr.	Heat and dampness, detoxification, cooling blood	Orally	Sealed storage
Dalitong Keli	Granules	*Bupleurum chinense* DC.; *Citrus aurantium*; *Saussurea costus*; *T. mongolicum* Hand.-Mazz; C*itrus reticulata* Blanco; *Paederia scandens* (Lour.) Merr.; *Corydalis yanhusuo* W. T. Wang	Clearing away heat, relieving depression, stomach down	Orally	Sealed and stored in a dry place
Fule Keli	Granules	*Lonicera japonica* Thunb.; *Sargentodoxa cuneata* (Oliv.) Rehder & E.H. Wilson; *T. mongolicum* Hand.-Mazz; *Isatis indigotica*; *Paeonia veitchii* Lynch; *Melia toosendan* Sieb. et Zucc.; *Corydalis yanhusuo* W. T. Wang	Clearing heat, cooling blood, relieving phlegm and pain	Orally	Sealed storage
Lianpu Shuangqing Pian	Troche	Berberine salt; Extract of *T. mongolicum* Hand.-Mazz;	Detoxification, relieving dryness and dampness	Orally	Sealed storage
Kangyan Tuire Pian	Troche	*Taraxacum mongolicum* Hand.-Mazz; *Scutellaria baicalensis* Georgi	Clearing away heat, detoxification, eliminating and dispersing swelling	Orally	Sealed and stored in a cool dry place
Kanggusuiyan Pian	Troche	*Lonicera japonica* Thunb.; *Viola yedoensis* Makino; *T. mongolicum* Hand.-Mazz; *Scutellaria barbata* D. Don; *Pulsatilla chinensis* (Bge.) Regel; *Hedyotis diffusa* Willd.	Clearing away heat, detoxification, dissipating swelling	Orally	Sealed storage
Nankang Pian	Troche	*Hedyotis diffusa* Willd.; *Scutellaria baicalensis* Georgi; *T. mongolicum* Hand.-Mazz; *Paeonia veitchii* Lynch; *Carthamus tinctorius* L.; *Angelica sinensis* (Oliv.) Diels; *Cuscuta chinensis* Lam.	Tonifying kidney and activating blood circulation, clearing heat, detoxification	Orally	Sealed storage
Libi Pian	Troche	*Scutellaria baicalensis* Georgi; *Xanthium sibiricum* Patr.; *T. mongolicum* Hand.-Mazz; *Magnolia biondii* Pamp.; *Angelica dahurica*	Detoxification	Orally	Sealed storage
Shenyan Jiere Pian	Troche	*Smilax glabra* Roxb.; *Imperata cylindrica* var. major (Nees) C. E. Hubb.; *T. mongolicum* Hand.-Mazz; *Cinnamomum cassia* (L.) J.Presl; *Forsythia suspensa* (Thunb.) Vahl; *Nepeta cataria* L.	Dispelling wind, relieving heat, diffusing the lungs	Orally	Sealed storage
Jinpu Jiaonang	Capsule	*Lonicera japonica* Thunb.; *T. mongolicum* Hand.-Mazz; *Scutellaria barbata* D. Don; *Curcuma zedoaria* (Christm.) Roscoe; *Boswellia carterii*; *Astragalus membranaceus var. mongholicus* (Bge.) Hsiao; *Codonopsis pilosula* (Franch.) Nannf.	Clearing away heat, detoxification, reducing swelling and relieving pain, benefiting Qi, dissipating phlegm	Orally	Sealed storage
Jinsang Sanjie Wan	Pill	*Strobilanthes cusia*; *Lonicera japonica* Thunb.; *T. mongolicum* Hand.-Mazz; *Scrophularia ningpoensis* Hemsl.; *Ophiopogon japonicus* (Thunb.) Ker-Gawler	Clearing away heat, detoxification, promoting blood circulation, removing blood stasis	Orally	Sealed storage
Rupixiao Pian	Troche	*T. mongolicum* Hand.-Mazz; *Trichosanthes kirilowii*; *Spatholobus suberectus*; *Aucklandia lappa* Decne.; *Prunella vulgaris* L.; *Carthamus tinctorius* L.	Activating blood circulation, removing blood stasis, clearing away heat and toxic substances	Orally	Sealed storage
Rupixiao Jiaonang	Capsule	*T. mongolicum* Hand.-Mazz; *Trichosanthes kirilowii*; *Aucklandia lappa* Decne.; *Panax notoginseng* (Burk.) F. H. Chen; *Paeonia veitchii* Lynch; *Carthamus tinctorius* L.; *Scrophularia ningpoensis* Hemsl.	Activating blood circulation, removing blood stasis, clearing away heat and toxic substances	Orally	Sealed storage
Rupixiao Keli	Granules	*T. mongolicum* Hand.-Mazz; *Trichosanthes kirilowii*; *Aucklandia lappa* Decne.; *Panax notoginseng* (Burk.) F. H. Chen; *Paeonia veitchii* Lynch; *Carthamus tinctorius* L.; *Scrophularia ningpoensis* Hemsl.	Activating blood circulation, removing blood stasis, clearing away heat and toxic substances	Orally	Sealed storage
Weichang Fuyuan Gao	Paste	*Pseudostellaria heterophylla* (Miq.) Pax ex Pax et Hoffm.; *Rheum palmatum* L.; *Raphanus sativus* L.; *T. mongolicum* Hand.-Mazz; *Aucklandia lappa* Decne.; *Astragalus membranaceus* var. mongholicus (Bge.) Hsiao	Replenishing qi, activating blood and qi	Orally	Sealed and stored in a cool place
Fufang Qingdai Wan	Pill	Indigo Naturalis; *Prunus mume* (Sieb.) Sieb. et Zucc.; *Arnebia euchroma* (Royle) Johnst.; *T. mongolicum* Hand.-Mazz; *Portulaca oleracea* L.; *Dictamnus dasycarpus* Turcz.	Clearing heat, cooling blood, detoxification, spotting	Orally	Sealed storage
Fufang Jinhuanglian Keli	Granules	*Forsythia suspensa* (Thunb.) Vahl; *Scutellaria baicalensis* Georgi; *Strobilanthes cusia; T. mongolicum* Hand.-Mazz; *Lonicera japonica* Thunb.	Clearing away heat, detoxification	Orally	Sealed and stored in a cool place
Fufang Zhenzhu Anchuang Pian	Troche	*Lonicera macranthoides* Hand.-Mazz; *T. mongolicum* Hand.-Mazz; *Scutellaria baicalensis* Georgi; *Phellodendron amurense* Rupr.; *Angelica sinensis* (Oliv.) Diels; *Glehnia littoralis* Fr. Schm. ex Miq.; Pearl; *Clematis armandii* Franch.	Clearing away heat, detoxification, cooling blood, eliminating erythema	Orally	Sealed storage
Fufang Yigan Wan	Pill	*Panax ginseng* C.A. Mey.; *Strobilanthes cusia*; *Chrysanthemum indicum* L.; *T. mongolicum* Hand.-Mazz; *Smilax glabra* Roxb.; *Plantago asiatica*; *Carthamus tinctorius* L.	Clearing away heat and dampness, soothing the liver and spleen, dissipating phlegm	Orally	Sealed storage
Fufang Huangbaiye Tuji	Liquid pharmaceutical preparations;	*Forsythia suspensa* (Thunb.) Vahl; *Phellodendron amurense* Rupr.; *Lonicera japonica* Thunb.; *Centipede*; *T. mongolicum* Hand.-Mazz	Detoxification, reducing swelling and rot	External	Sealed and stored in a cool place
Danshitong Jiaonang	Capsule	*T. mongolicum* Hand.-Mazz; *Artemisia capillaris* Thunb.; *Lysimachia christinae* Hance; *Bupleurum chinense* DC.; *Rheum palmatum* L.; *Scutellaria baicalensis* Georgi; *Citrus aurantium* L.	Clearing away heat and dampness, cholagogic, stone removing	Orally	Sealed storage
Dankang Jiaonang	Capsule	*Bupleurum chinense* DC.; *Rheum palmatum* L.; *Curcuma aromatica* Salisb.; *T. mongolicum* Hand.-Mazz; *Artemisia capillaris* Thunb.; *Gardenia jasminoides* Ellis; *Mentha haplocalyx* Briq.	Soothing liver, cholagogic, clearing away heat and toxins, relieving inflammation and pain	Orally	Sealed and protected from moisture
Qianlietong Pian	Troche	*Vaccaria segetalis* (Neck.) Garcke; *Plantago asiatica*; *Astragalus membranaceus var. mongholicus* (Bge.) Hsiao; *T. mongolicum* Hand.-Mazz; *Lycopus lucidus var. hirtus* Regel; *Illicium verum* Hook. f.; *Cinnamomum cassia* Presl	Clearing away dampness and turbidity, removing blood stasis, dispersing stagnation	Orally	Sealed storage
Reyanning Pian	Troche	*T. mongolicum* Hand.-Mazz; *Reynoutria japonica* Houtt.; *Ixeris polycephala*; S*cutellaria barbata* D. Don	Detoxification	Orally	Sealed storage
Reyanning Heji	Liquid pharmaceutical preparation	*T. mongolicum* Hand.-Mazz; *Reynoutria japonica* Houtt.; *Ixeris polycephala*; S*cutellaria barbata* D. Don	Detoxification	Orally	Sealed and stored in a cool place
Reyanning Keli	Granules	*T. mongolicum* Hand.-Mazz; *Reynoutria japonica* Houtt.; *Ixeris polycephala*; *Scutellaria barbata* D. Don	Detoxification	Orally	Sealed storage
Langchuang Wan	Pill	*Lonicera japonica* Thunb.; *Forsythia suspensa* (Thunb.) Vahl; *Coptis chinensis* Franch.; *T. mongolicum* Hand.-Mazz; *Rehmannia glutinosa* (Gaertn.) Libosch. ex Fisch. & C.A. Mey.; *Paeonia veitchii* Lynch; *Scrophularia ningpoensis* Hemsl.	Clearing away heat, detoxification, cooling blood, promoting blood circulation	Orally	Sealed storage
Xiaoyan Tuire Keli	Granules	*Isatis indigotica*; *Viola yedoensis* Makino; *Glycyrrhiza uralensis* Fisch. ex DC.; *T. mongolicum* Hand.-Mazz	Clearing away heat and detoxifying, cooling blood, reducing swelling	Orally	Sealed storage
Xiaocuo Wan	Pill	*Cimicifuga foetida* L.; *Bupleurum chinense* DC.; *Ophiopogon japonicus* (Thunb.) Ker-Gawler; *T. mongolicum* Hand.-Mazz; *Scrophularia ningpoensis* Hemsl.; *Dendrobium nobile* Lindl.; *Prunella vulgaris* L.	Clearing away heat and dampness, detoxification, dispersing	Orally	Sealed storage
Sangge Jiangzhi Wan	Pill	*Taxillus chinensis* (DC.) Danser; *Pueraria lobata* (Willd.) Ohwi; *Dioscorea opposita* Thunb.; *T. mongolicum* Hand.-Mazz; *Crataegus pinnatifida* Bunge; *Salvia miltiorrhiza* Bunge; *Alisma orientalis* (Sam.) Juzep.	Invigorating the kidney, strengthening the spleen, dissipating phlegm, clearing away heat and dampness	Orally	Sealed storage
Yinpujiedu Pian	Troche	*Lonicera macranthoides* Hand.-Mazz; *Chrysanthemum indicum* L.; Prunella vulgaris L.; *T. mongolicum* Hand.-Mazz; *Viola philippica* Cav.	Detoxification	Orally	Sealed storage
Kangfu Xiaoyan Shuan	Suppository	*Viola philippica* Cav.; *Sophora flavescens* Aiton; *Patrinia scabiosaefolia* Fisch.; *Andrographis paniculata* (Burm. f.) Nees; *T. mongolicum* Hand.-Mazz; A*rnebia euchroma* (Royle) Johnst.	Clearing away heat, detoxification, dampness and dispersing, killing insects and itching	Rectal administration	Sealed and stored in a cool place
Pudilan Xiaoyan Koufuye	Liquid pharmaceutical preparation	*T. mongolicum* Hand.-Mazz; *Strobilanthes cusia*; *Scutellaria baicalensis* Georgi; *Corydalis bungeana*	Clearing away heat, detoxification, reducing swelling	Orally	Sealed storage

**Table 2:** Use of *Taraxacum* spp. plants in traditional medicine.

Plant Species	Functions	Countries	References
*T. androssovii* Schischkin	Wounds, stomach disorders	Turkey	[8]
*T. cyprium H.* Lindb.	Anti-cough, expectorant, dyspepsia	Cyprus	[9]
*T. fedtschenkoi* Hand.- Mazz.	Wounds, stomach disorders	Turkey	[8]
*T. macrolepium* Schischkin	Wounds, stomach disorders	Turkey	[8]
*T. mongolicum* Hand.-Mazz	Tuberculosis, fever, acne	China	[10]
*T. oellgaardii* C. C. Haw. synonym, *T. officinale* (L.) Weber ex F. H. Wigg	Fever, cough, dysmenorrhea, headache, constipation, stomach, pain, digestive, stomachache, skin problems, toothache, wounds, swelling, digestive disorders, peptic ulcer, migraine, abdominal complaints, blisters and rash, treatment of gastrointestinal, diseases, eczema	India, Mexico, Pakistan, Kosovo, Romania, Bulgaria, Argentina, Italy, Serbia, Bolivia, Georgia, Peru, Turkey	[11,12,13,14,15,16,17,18,19,20,21,22,23,24,25,26,27,28,29]
*T. platycarpum* Dahlst.	Furuncles	Korea	[30]
*T. Stevenii* (Spreng.) DC.	Toothache, abdominal spasms	Turkey	[31]

**Table 3:** The bioactivity of extracts from different species of *Taraxacum*.

Extracts/Compounds	Species	Formulation/Dosage	Results	References
**Anti-bacteria**				
Ethanol extracts	*T. mongolicum*	In vitro; 1 g·mL^−1^	*T. mongolicum* has a higher nutritional value, better antimicrobial effects and is an edible plant.	[78]
Ethanol extracts from flowers	*T. mongolicum*	In vitro; Gram-negative bacteria (125 to 250 μg·mL^−1^) and Gram-positive bacteria (62.5 to 250 μg·mL^−1^)	The anti-bacterial test results showed that this fraction strongly inhibited the growth of all of the microorganisms, especially *P. aeruginosa* and *B. subtilis* (with MIC values of 125 μg·mL^−1^ and 62.5 μg·mL^−1^, respectively)	[79]
Aqueous and ethanol extracts	*T. mongolicum*	In vitro; 0.125, 0.25, 0.5 g·mL^−1^	It could inhibit these bacteria at different level in which ethanolic extracts of *P. oleracea* L. generally had higher anti-bacterial activities than aqueous extracts.	[66]
Water extracts	*T. mongolicum*	In vivo; 20 mg·kg^−1^	The possibility of a multifactorial drug−drug interaction existed between extracts and ciprofloxacin. Thus, the implications of concomitant dosing of the two agents should not be overlooked.	[80]
Extracts from leaves	*T. officinale*	In vitro	It was found to be effective against all the tested Bacterial pathogens *P. aeruginosa*, *E. coli, S. aureus, B. Subtilis* and *M. luteus*.	[81]
Extracts from roots	*T. officinale*	In vitro	It exhibited considerable α-amylase and α-glucosidase inhibitory activities.	[82]
Extracts from leaves	*T. officinale*	In vitro	It displayed excellent antimicrobial activity against *S. aureus* and *E. coli.*	[83]
Peptides	*T. officinale*	In vitro	It displayed high antimicrobial activity both against fungal and bacterial pathogens.	[84]
Ethanol extracts from leaves	*T. officinale*	In vitro	It had shown an antimicrobial activity against the bacterial strains of *E. coli* and *S. abony*, but had not shown any antimicrobial activity against *S. aureus.*	[85]
Endophytic fungi	*T. coreanum*	In vitro	The results indicated that the endophytic fungis had the ability to antifungal.	[86]
**Anti-oxidant**				
Methanol extracts	*T. coreanum*	In vitro	Its anti-oxidant activity was presented in a dose-dependent pattern.	[87]
Extracts	*T. officinale*	In vitro	It inhibited oxidative stress through elevated de novo synthesis of anti-oxidative enzymes and suppression of iNOS expression by NF-B inactivation.	[88]
Methanol extracts	*T. mongolicum*	In vitro	The anti-oxidant activity of *T. mongolicum* was presented in a dose-dependent pattern.	[89]
Extracts	*T. officinale*	In vitro	Dandelion root was a valuable source of dietary fibers and natural anti-oxidants.	[90]
Ethanol extracts	*T. officinale*	In vivo; 50,100, and 200 mg·kg^−1^ (20 days)	The study indicated efficacy of dandelion extract on RBC (group) and HB (group) in doses of 50,100, and 200 mg·kg^−1^ and in 200 mg·kg^−1^ on WBC (group) to achieve normal body balance.	[91]
Methanol extracts	*T. officinale*	In vitro	Its anti-oxidant activity was presented in a dose-dependent pattern.	[87,89]
Methanol extracts	*T. obovatum*	In vivo and in vitro	The results found the extracts to be the most promising species with anti-oxidative capacity and only *T. lacistrum* to present reliable cytotoxicity over HeLa and HepG2 cell lines, with an interesting SI. A proper species determination, using its distribution or deep botanical description, was required for plants of the genus *Taraxacum,* as pharmacological abilities mainly vary between species.	[92]
Methanol extracts	*T. marginellum*	In vivo and in vitro		
Methanol extracts	*T. hispanicum*	In vivo and in vitro		
Methanol extracts	*T. lambinonii*	In vivo and in vitro		
Methanol extracts	*T. lacistrum*	In vivo and in vitro		
Ethanol from flowers	*T. officinale*	In vitro; 50, 100, 150 μg·mL^−1^	The prevention of living cells from peroxyl radical-induced oxidation in the presence of dandelion flower extract suggested that the standardized extract had biological anti-oxidant activity.	[93]
Extracts	*T. mongolicum*	In vitro	The extracts suppressed the damage to osteoblasts under oxidative stress and are potential anti-oxidant materials for preventing bone diseases.	[94]
Methanol extracts	*T. mongolicum*	In vivo and in vitro	The extracts had significantly inhibitory activities on monoamine oxidase-A/B.	[95]
Ethanol extracts from the roots and leaves	*T. officinale*	In vitro; 400, 500, and 600 µg·mL^−1^	The extracts showed effective anti-oxidant activity correlating with total flavonoid and polyphenol contents.	[96]
Ethanol extracts from fruit	*T. officinale*	In vivo; 1, 5, 10, and 20 μg·mL^−1^	The extracts protected against SNP-induced decreases in cellular viability and increased in lipid peroxidation in the cortex, hippocampus, and striatum of rats.	[97]
Ethanol extracts from leaves	*T. officinale*	In vivo; 0.1, 0.5 mg·kg^−1^	The results clearly demonstrated the hepatoprotective effect of extracts against the toxicity induced by acetaminophen.	[98]
Granules of leaves and roots	*T. officinale*	In vivo; 250 g·day^−1^ (4 weeks)	The treatment with dandelion root and leaf positively changed plasma anti-oxidant enzyme activities in cholesterol-fed rabbits.	[99]
Methanol extracts	*T. sect. Ruderalia*	In vitro	The vegetative parts gave higher anti-oxidant activity, which could be related to its higher content in phenolic acids.	[32]
Extracts from flowers	*T. officinale*	In vitro; 0, 0.5, 1.0, and 2.5 µg·mL^−1^	The extracts possessed both anti-oxidant and cytotoxic properties which could, in part, be attributed to the presence of luteolin and luteolin 7-glucoside.	[93,100]
**Anti-cancer**				
Methanol extracts from shoots/roots	*T. coreanum*	In vitro 200, 400 mg·kg^−1^	Calu-6; IC_50_ = 140.2/101.6 mg·kg^−1^	[87]
Methanol extracts from shoots/roots	*T. mongolicum*	In vitro 200/400 mg·kg^−1^	Calu-6; IC_50_ = 83.4/66.4 mg·kg^−1^	[87]
Methanol extracts from shoots/roots	*T. officinale*	In vitro 200, 400 mg·kg^−1^	Calu-6; IC_50_ = 165.6/978.4 mg·kg^−1^	[87]
Ethanol extracts	*T. mongolicum*	In vitro	It induced G2/M phase arrest and activated apoptosis in MDA-MB-231 cells through ER stress.	[57]
Methanol–water extracts from roots	*T. japonicum*	In vivo	An extract of the roots of the plant could be a valuable chemopreventive agent against chemical carcinogenesis.	[101]
Flavonoids extraction	*T. officinale*	In vitro	The anti-oxidant activity of the purified flavonoids displayed strong ablilty.	[102]
Extracts	*T. officinale*	In vitro; 2–0.02 mg·mL^−1^	The extracts induced cytotoxicity through TNF-α and IL-1α secretion in HepG2 cells.	[103]
**Anti-inflammatory**				
Ethanol extracts	*T. officinale*	In vivo; 50, 100 and 200 mg·kg^−1^	The extract possessed acute anti-inflammatory activity.	[104]
Polysaccharides	*T. officinale*	In vivo; 304, 92 mg·kg^−1^ (7 days)	The polysaccharides had a hepatoprotective effect by modulating inflammatory responses and ameliorating oxidative stress.	[105]
Ethanol extracts	*T. coreanum*	In vitro and in vivo; 0, 10, 25, 50, 100, 200, and 400 mg·mL^−1^	The extracts possessed potent anti-inflammatory activity in vitro and in vivo, which occurred at least partly through inhibition of pro-inflammatory signaling and mediator release.	[106]
Chloroform extracts	*T. officinale*	In vitro	The fraction significantly suppressed production of NO, PGE2, and two pro-inflammatory cytokines (TNF-α and IL-1β) in a dose-dependent manner with 50% inhibitory concentration values of 66.51, 90.96, 114.76, and 171.06 μg·mL^−1^, respectively.	[107]
Water extracts	*T. mongolicum*	In vitro; 10, 100, 1000 μg·mL^−1^	Treatment of extracts significantly inhibited NO production in LPS-stimulated MACT cells.	[108]
Water extracts	*T. mongolicum*	In vivo	*T. mongolicum* could exert some of its anti-inflammatory and pharmacological effects by affecting the activity of PI3K/Akt/mTOR in LPS-induced acute lung injury in mice.	[69]
Organic acid	*T. mongolicum*	In vivo; 5 mg·kg^−1^	Organic acid could improve LPS-induced histopathological damage of tracheal tissues through the regulation of TLR4/NF-κB and TLR4/IKK/NF-κB signaling pathways and could be beneficial for the treatment of acute tracheobronchitis.	[72,73]
Methanol extracts	*T. officinale*	In vitro; 50, 100, 200, 400 μg·mL^−1^	The results of RAW 264.7 macrophage cells indicated the extracts had excellent anti-inflammatory effects.	[109]
Methanol extracts	*T. hallaisanense*			
Methanol extracts	*T. ohwianum*			
Methanol extracts	*T. coreanum*			
Methanol extracts	*T. platycarpum*			
Ethanol extracts	*T. officinale*	In vitro	The extracts possessed marked anti-inflammatory activity.	[104]
**Other effects**				
Methanol extracts	*T. mongolicum*	In vitro; 5, 10, 50, 100, 500, 1000 μg·mL^−1^	By the results of cytotoxicity of TAM on the B16F10 cell, little cytotoxicity was exhibited from every concentration from 5 g·mL^−1^ to 1000 g·mL^−1^.	[110]
1β,3β-dihydroxy-eudesman-11(13)-en-6α,12-olide	*T. mongolicum*	In vitro	This compound was found to have an inhibitory activity on nitric oxide production with an IC_50_ of 38.9 µM in activated RAW 264.7 cells.	[36]
1β,3β-dihydroxyeudesman-6α,12-olide	*T. mongolicum*	In vitro	This compound was found to have an inhibitory activity on nitric oxide production with an IC_50_ of 32.4 µM in activated RAW 264.7 cells.	[36]
Water extracts	*T. mongolicum*	In vitro	The results showed no significantly cytotoxic effects on the MAC-T cells at 1–1000 μg·mL^−1^ of extracts.	[108]
Ethanol extracts	*T. mongolicum*	In vitro; 0, 50, 100, 200, and 400 mg·mL^−1^	It possessed the most effective hypolipidemic activity in HepG2 cells.	[111]
Chlorogenic acids	*T. antungense*	In vitro	TaHQT1 and TaHQT2 function in the biosynthesis of 5-caffeoylquinic acid, but the genes showed tissue-specific expression patterns, suggesting a mechanism for the regulation of 5-caffeoylquinic acid production.	[112]
Ethanol extracts	*T. mongolicum*	In vitro	The results demonstrated the potential estrogenic activities of the extract, providing scientific evidence supporting their use in traditional medicine.	[70]
Ethanol extracts	*T. mongolicum*	In vitro	The extracts at 50–100 µg·mL^−1^ improved D-galactosamine, thioacetamide and tert-butyl hydroperoxide (t-BHP)-injured rat hepatocytes, and produced protection rates of 42.2, 34.6, and 43.8% at 100 µg·mL^−1^, respectively.	[71]
Water–ethanol extracts from roots	*T. officinale*	In vivo; 200, 600 mg·kg^−1^ (10 days)	Hepatic Cu/Zn SOD activity decreased in intoxicated mice and normalized in extract-treated groups.	[113]
Extracts	*T. officinale*	In vivo; 50 mg·kg^−1^ (30 days)	The body weight of mice and rats was decreased after administration of extracts.	[114]
Extracts	*T. officinale*	In vivo; 100 mg·kg^−1^ (20 days)	The extracts which were used against histopathological changes in the kidney caused by toxication showed a corrective effect, which were supported by biochemical parameters.	[115]
Water extracts of leaves	*T. officinale*	In vitro; 25 mg·kg^−1^ (14 days)	The study revealed that leaf extract could afford a significant protection against CCl4-induced hepatocellular injury.	[116]
Extracts of leaves	*T. officinale*	In vitro; 0.2 g·mL^−1^	The methylene chloride inhibited as much as 97% of proliferation of the SGT cells and only about 7% of the RAW 246.7 cells. Ethyl acetate and butanol fractions inhibited 42.03% and 24.35% proliferation of the SGT cells, respectively, and only 12% and 8% of the RAW 246.7 cells.	[83]
Taraxinic acid	*T. coreanum*	in vitro	The induction of HL-60 cell maturation by taraxinic acid may have potential as a therapeutic approach for the treatment of leukemia.	[117]
Methanol extracts	*T. platycarpum*	In vitro	The triterpene fraction had an effect on the proliferation of normal skin fibroblasts at a concentration of 10 or 5.0 μg·mL^−1^, but some compounds showed cytotoxicity and anti-proliferative activity toward fibroblasts at the same concentration.	[47]
The water extracts from roots and leaves	*T. officinale*	In vivo; 50, 100, and 200 mg·kg^−1^	The results clearly demonstrated the antidepressant effects of extracts in animal models of behavioral despair and suggested the mechanism involved in the neuroendocrine system.	[118]
Methanol extracts from leaves	*T. officinale*	In vivo; 150, 300 mg·kg^−1^	The results revealed that leaf extracts had protective effects against CCl_4_- induced liver toxicity and damage.	[119]
Granules of leaves and roots	*T. officinale*	In vivo; 250 g·day^−1^ (4 weeks)	The treatment with dandelion root and leaf positively changed lipid profiles in cholesterol-fed rabbits.	[99]
Ethanol extracts	*T. officinale*	In vivo; 1 g·mL^−1^ (1 day)	It showed promising potential as a diuretic in humans.	[120]
Extracts from leaves	*T. officinale*	In vivo; 2 g·kg^−1^ (10 weeks)	The extracts may represent a promising approach for the prevention of high-fat diet-induced nonalcoholic fatty liver.	[121]
Root extracts	*T. officinale*	In vivo; 250, 500, 750 mg·kg^−1^	The administration of extracts ameliorated CCl_4_ induced liver damage.	[122]
Desacetylmatricarin	*T. platycarpum*	In vitro	The results showed a potent inhibitory activity upon the β-hexosaminidase release from RBL-2H3 cells in a dose-dependent manner and the IC_50_ was 7.5 μM.	[48]
Extracts	*T. officinale*	In vivo; 100 mg·kg^−1^	*n*-Butanol fraction-induced increase in gastric emptying was related to smooth muscle contraction.	[123]
Water extracts	*T. officinale*	In vivo; 2.5, 5, and 10 mg·kg^−1^	The extracts protected against lipopolysaccharide-induced acute lung injury in mice.	[124]
Ethanol extracts	*T. officinale*	In vivo; 150 mg·kg^−1^ (1 week)	The extracts exhibited hepatoprotective activity in CCl_4_- induced hepatic damage in mice.	[125]
Water extracts	*T. officinale*	In vivo; 10, 100 mg·kg^−1^ (10 days)	The extracts improved fatigue-related indicators and immunological parameters in mice.	[126]

## Data Availability

Not applicable.

## Abbreviations

COX-2: cyclooxygenase-2; D-GalN: D-galactosamine; HPLC: high-performance liquid chromatography; HSCCC: high-speed counter-current chromatography; IL-1β: interleukin-1β; iNOS: inducible NO synthase; LPS: lipopolysaccharide; MIC: minimum inhibitory concentration; MTT: tetrazolium salt colorimetric; NMR: nuclear magnetic resonance; PG: prostaglandin; PHPLC: preparative HPLC; TAA: thioacetamide; t-BHP: t-butyl hydroperoxide; TCM: traditional Chinese medicine; TNBC: triple-negative breast cancer; TNF-α: tumor necrosis factor-α; UPLC: ultra-performance liquid chromatography.
